# Stab wound of the superficial femoral artery early diagnosed by point-of-care Doppler ultrasound

**DOI:** 10.1186/s13089-020-00179-2

**Published:** 2020-06-16

**Authors:** Pablo Blanco, María Fernanda Menéndez

**Affiliations:** 1 Intensive Care Unit, Clínica Cruz Azul, 2651, 60 St, Necochea, 7630 Argentina; 2 Department of Teaching and Research, Hospital “Dr. Emilio Ferreyra”, 4801, 59 St, Necochea, 7630 Argentina; 3 Intensive Care Unit, Hospital “Dr. Emilio Ferreyra”, 4801, 59 Ave, Necochea, 7630 Argentina

**Keywords:** Point-of-care, Doppler ultrasound imaging, Trauma, Vascular injuries

## Abstract

**Background:**

Traumatic vascular injury of the limbs has the potential to cause substantial patient morbidity and mortality, and therefore, early recognition and treatment are crucial to improve outcomes. While patients with hard signs of arterial injury mandate for an immediate surgical intervention, patients presenting with soft signs of arterial injury need further diagnostic evaluation.

**Case presentation:**

A 24-year-old male was admitted to the emergency department after suffering a stab wound in the anterolateral aspect of his left upper thigh. Entry wound measures approximately 3 cm × 0.7 cm; no exit wound was observed. On examination of the injured limb, the thigh was swollen and painful. Skin color was mildly pale and skin temperature was slightly diminished in his leg; leg numbness was also pointed out by the patient. Common femoral artery pulse was normal, while distal pulses were diminished. Point-of-care Doppler ultrasound (DUS) showed a subfascial hematoma in the thigh, which filled on color Doppler, corresponding to a pseudoaneurysm. On spectral Doppler, signs of distal low blood supply were noted. The patient was immediately transferred to the operating room where a 1-cm laceration was found in the anterior aspect of the superficial femoral artery. The involved artery was successfully repaired and distal flow was reestablished, as assessed by clinical examination, pulse palpation and DUS.

**Conclusions:**

Based on its several advantages, DUS should be considered as the first-line diagnostic tool in the diagnostic workup of patients with soft signs of arterial injury.

## Introduction

Vascular injuries represent less than 3% of all traumatic findings and have a potential to cause morbidity and mortality if they are not timely recognized and treated [1–4]. The majority of vascular injuries occur in the extremities and may result from penetrating trauma, blunt trauma, or both. Among them, gunshot injury is the leading cause, followed by stab wounds and blunt trauma [1]. Mortality is mainly related to hypovolemia after a major vessel is injured, while morbidity may result secondary to compartment syndrome, arteriovenous fistula, limb loss or wound infections [1–4].

Vascular injury can be arterial, venous or mixed [2]. Serious arterial injury may result from transection, laceration or dissection while venous injury is mainly related to vein rupture or thrombosis and rarely from venous wall dissection [2].

Among patients with arterial injury, those presenting with “hard signs” of arterial injury (e.g., pulsatile bleeding, expanding hematoma, absent distal pulses, cold/pale limb, palpable thrill or an audible bruit) mandate for an immediate surgery and vascular repair without the support of diagnostic imaging techniques. On the other hand, patients with “soft signs” of arterial injury such as diminished pulses, small and non-expanding hematoma or proximity of wound to a major vessel require further diagnostic evaluation [1, 5], such as the ankle-brachial pressure index (ABPI), angiography, or Doppler ultrasound (DUS).

We present hereby the case of a patient with stab injury of the superficial femoral artery demonstrated by point-of-care DUS and how the use of this method impacted in decision-making.

## Case report

**Fig. 1:**
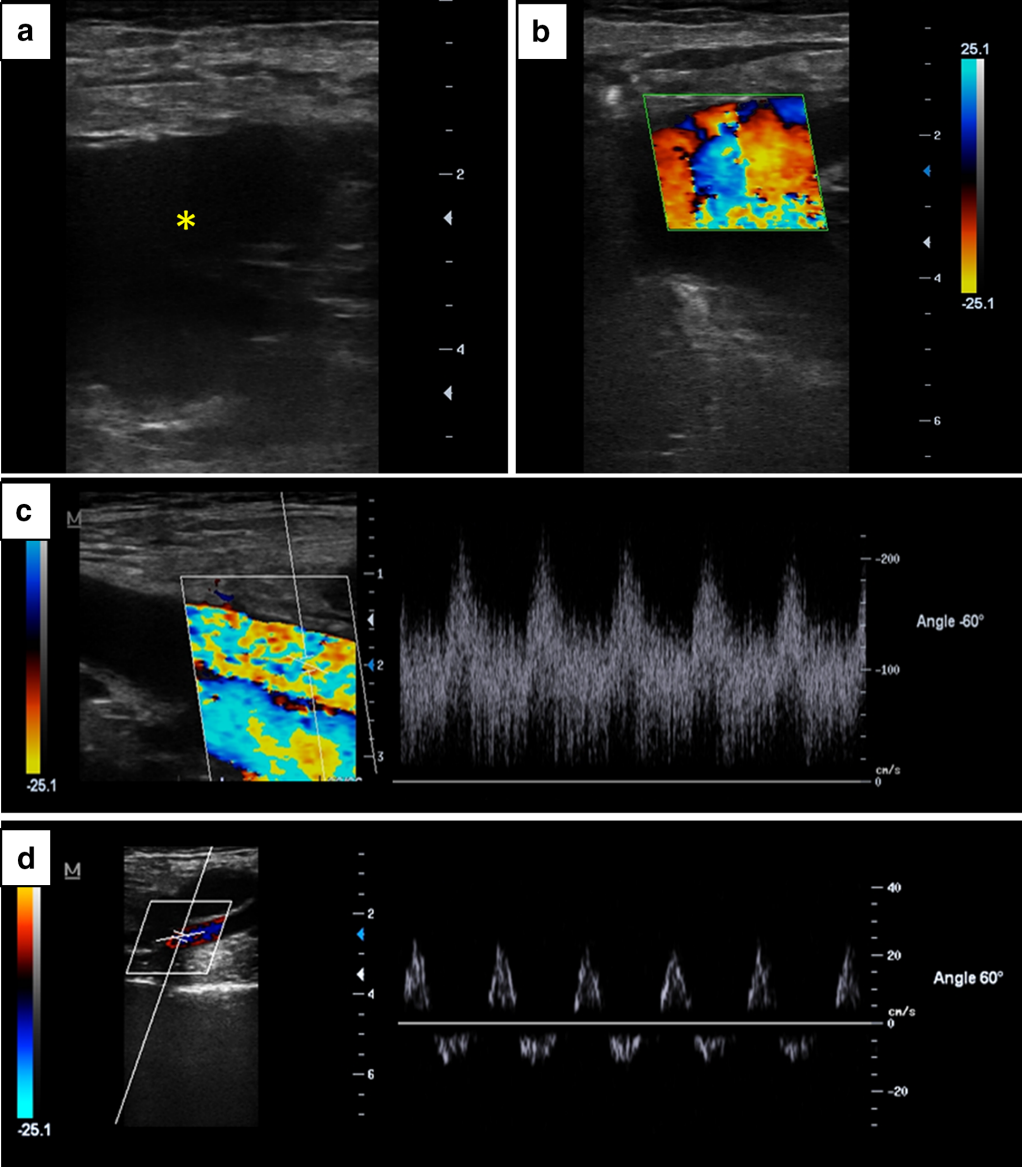
Stab wound of the left-sided superficial femoral artery. **a** Subfascial fluid collection corresponding to a hematoma (asterisk); **b** Color Doppler demonstrating flow in the hematoma, corresponding to a pseudoaneurysm; **c** spectral Doppler of the superficial femoral artery proximal to the site of injury, showing high-velocity monophasic waveforms, indicating a reduction in the vascular resistance distal to the insonation site (arterial laceration) and compensatory flow; **d** spectral Doppler in arteries distal to the site of injury, showing low-velocity biphasic waveforms, indicating low blood supply

A 24-year-old male was brought to the emergency department (ED) immediately after suffering a stab injury in his left thigh which he alleged to be produced during a neighborhood altercation. A kitchen knife entered from the anterolateral aspect of the upper third of his left thigh, leaving a 3 cm × 0.7 cm entry wound which penetrates into the deep tissues; no exit wound was found. On admission, tachycardia (120 beats/min) and mild arterial hypotension (80/50 mmHg) were observed. On the injured limb, physical examination showed a painful and swollen tight. He was able to move the entire limb; however, he noted numbness of his leg. Below the knee, skin color was mildly pale and temperature was slightly diminished. A prolonged capillary refill time was observed in his foot. Common femoral artery pulse was normal, while diminished pulses were palpated in popliteal, tibial anterior and tibial posterior arteries. A point-of-care DUS showed a large hypoechoic slightly pulsating hematoma in the subfascial compartment (Fig. 1a), which filled with a turbulent flow on color Doppler (Fig. 1b), consistent with a pseudoaneurysm. On spectral Doppler, common femoral artery and superficial femoral artery proximal to the hematoma showed a high velocity low-resistance profile (Fig. 1c), while popliteal, tibial posterior and tibial anterior arteries showed low-velocity biphasic waveforms (Fig. 1d), consistent with a proximal blood leak and reduced distal blood supply. Common femoral and femoral veins were patent and compressible.

**Fig. 2:**
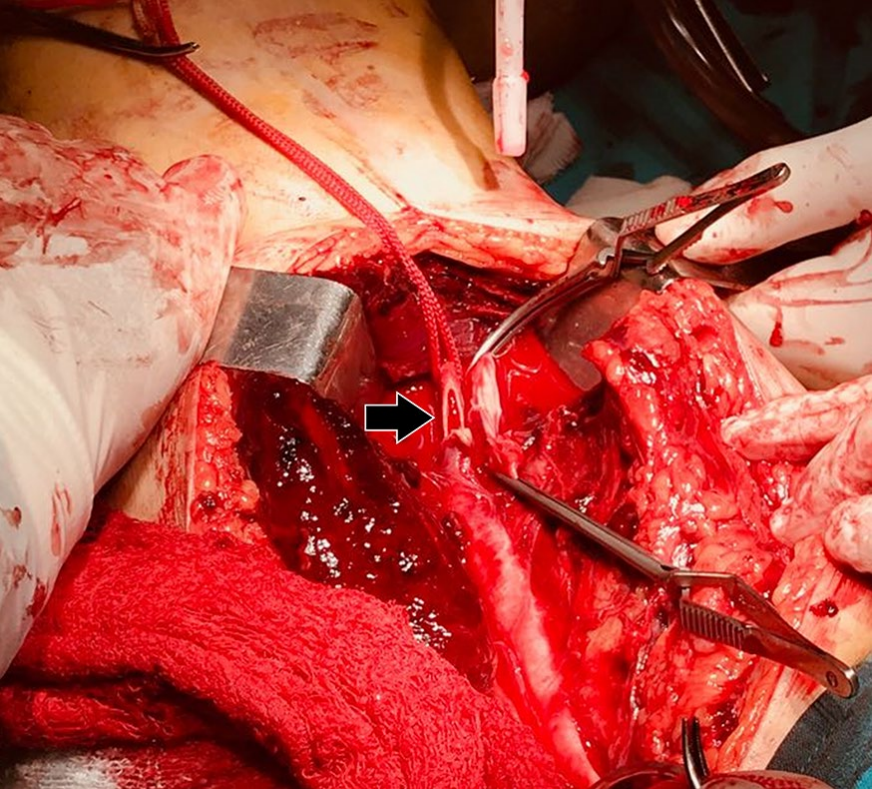
Surgery demonstrating a 1-cm laceration in the anterior aspect of the superficial femoral artery (arrow)

**Fig. 3:**
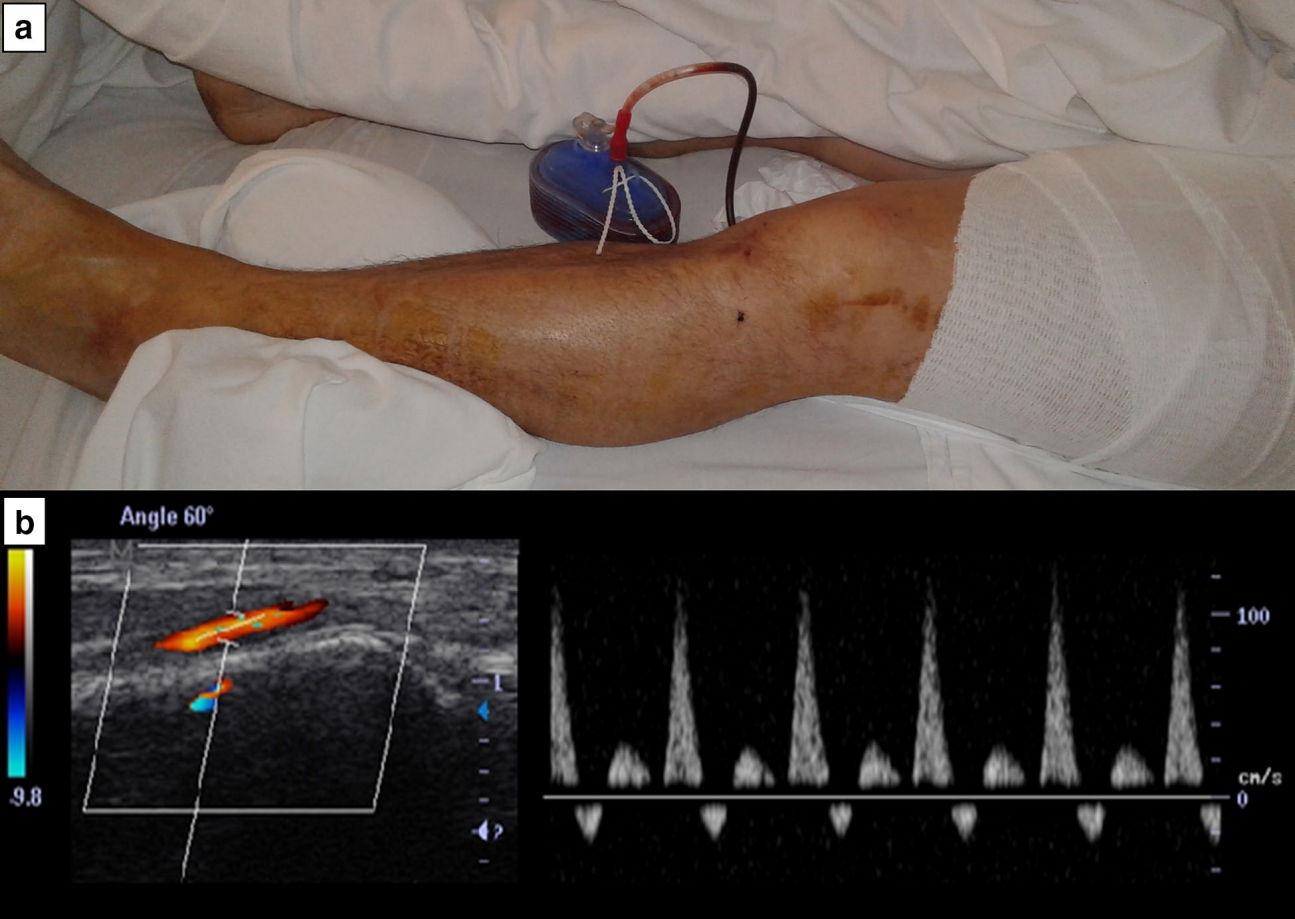
Postoperative care. **a** The patient’s left lower limb shows normal perfusion; **b** spectral Doppler shows normal-velocity triphasic waveforms in proximal and distal arteries (tibial posterior artery is only shown)

Given that arterial injury sounded unequivocal based on clinical examination and DUS findings, the patient was immediately transferred to the operating room for surgical exploration. A 1-cm longitudinal laceration was found in the anterior aspect of the superficial femoral artery (Fig. 2); deep veins were not compromised. The injured vessel was successfully repaired and then the patient was transferred to the intensive care unit for postoperative care. On the next day, skin color and temperature of the affected limb were normal, the patient indicated disappearance of his leg numbness, and distal pulses were full. DUS showed normal velocity triphasic waveforms in the proximal and distal arteries (Fig. 3). Patient follow-up was uneventfully and he was discharged from the hospital on day 5.

## Discussion

There are several methods varying in availability, complexity and costs for evaluating the patient with suspicion of arterial injury.

The ankle-brachial pressure index (ABPI), a simple, non-invasive, inexpensive and easy to perform vascular test, is considered by some authors as the first-line diagnostic tool for patients with “soft signs” of arterial injury of the lower limbs [5]. An ABPI > 1 practically excludes an arterial injury, while an ABPI < 1 warrants further investigation, such as DUS or angiography [5]. In the upper limbs, the wrist-brachial index can be used, with the same cutoff described for the ABPI. However, for practitioners working in emergency settings, the ABPI is poorly known, is not widely available and thus it is rarely used in this scenario.

Conventional angiography, which is considered the gold standard, or currently computed tomography angiography (CTA) or magnetic resonance angiography (MRA) aid in providing a definite diagnosis. However, these methods require transferring the patient to the Cath lab or the radiology department, use iodinated contrast media and ionizing radiation (CTA), or paramagnetic contrast media (MRA), and are expensive.

In contrast to the aforementioned techniques, DUS is nowadays widely available in the emergency department, is non-invasive, does not emit ionizing radiation and has demonstrated a good diagnostic accuracy for the diagnosis of vascular injury. Regarding the latter, the reported sensitivity of DUS is 95–97%, specificity of 95–98% and accuracy of 98% in assessment of peripheral vascular injuries [2]. A recent study showed a sensitivity and specificity of 100% to rule out a severe arterial injury when a triphasic flow was present [6]; the detection of other patterns (e.g., biphasic, monophasic flows) points toward the need of further investigations (see below). Two-dimensional imaging may show signs of vascular injury, such as a pseudoaneurysm, as seen in our patient, an intimal flap in the case of arterial dissection, or a partial or complete arterial thrombus [2]. Color Doppler may demonstrate a diminished or even the absence of flow, flow in the true and false lumen (arterial dissection) or the filling of the pseudoaneurysm (as shown in our presented case) [2]. In addition to two-dimensional and color Doppler imaging, spectral Doppler findings are of paramount importance. Normal arterial waveforms of the resting limbs typically show a triphasic pattern: the first corresponds to the rapid antegrade flow reaching a peak during systole; the second to a transient reversal of flow during early diastole which is secondary to the high vascular resistance imposed by the resting muscles, and the third to a slow antegrade flow during late diastole, resulting from wall recoil [2, 6–8]. As mentioned above, as a normal triphasic flow is detected, a severe arterial injury may be ruled out [6]. While biphasic, monophasic or even an absent flow is expected in patients with a severe acute arterial injury, changes in the spectral waveforms may be observed in several other circumstances as well [2, 6–8]. For example, given that the arterial wall becomes more rigid with advancing years, the third component is often lost in old-aged people. Other conditions include non-resting limbs, chronic arterial stenosis/occlusion and collateral circulation or infections (e.g., cellulitis). For that reasons, specificity of DUS for detecting a severe arterial injury is not guaranteed and further investigations should be considered when a non-triphasic flow is observed. Other aspect that is often assessed with DUS is flow velocity. As a rule of thumb, normal arterial peak systolic velocities (PSV) are around 100 cm/s in proximal arterial segments (e.g., common femoral artery), while distal segments show velocities of about 50 cm/s (e.g., posterior tibial artery) [8]. Low flow velocities or even an absent flow is expected in severe arterial injury. Similar to spectral waveforms, several factors may impact velocities, such as technical factors (wrong angle between flow and ultrasound beam), arterial hypo/hypertension, non-resting limbs or chronic arterial stenosis/occlusion. Given that interpretation of low flow velocities may be cumbersome in practice, it seems more reliable to base the DUS diagnosis on spectral waveforms (triphasic vs non-triphasic flow/no flow).

While a full lower limb DUS scan is time consuming and is nowadays reserved to radiologists, a focused DUS examination is desirable in point-of-care settings, as is the FAST D protocol, where a two-point DUS is proposed (posterior tibial artery and dorsalis pedis artery) [6]. While promising, the feasibility and reliability of the FAST D protocol in the hands of emergency or critical care physicians should be assessed in further studies.

As was demonstrated in our presented case, an abnormal flow pattern was clearly demonstrated, indicating low blood supply distal to the site of injury. High-velocity monophasic waveforms proximal to the injury site, as observed in our patient, may be explained by the arterial tear which lowers the vascular resistance distal to the insonated artery, and compensatory flow.

## Conclusions

DUS should be considered as the first-line method to detect a vascular injury of the limbs for the patient with soft signs of arterial injury in the emergency department. When there is suspicion of arterial injury, DUS aids in decision making, ruling out a severe arterial injury when triphasic waveforms are present; and pointing out to perform further investigations when non-triphasic waveforms/no flow is detected. Serial POCUS DUS examinations aid for the patient’s follow-up as well.

## Data Availability

Not applicable.

## Abbreviations

ABPI: Ankle-brachial pressure index
DUS: Doppler ultrasound
CTA: Computed tomography angiography
MRA: Magnetic resonance angiography
PSV: Peak systolic velocity
